# Neuropancreatology: The Nervous System and Pain Management in Pancreatic Diseases

**DOI:** 10.3390/life14030299

**Published:** 2024-02-23

**Authors:** Alberto Nicoletti, Federica Vitale, Mattia Paratore, Giuseppe Quero, Marcantonio Negri, Enrico Celestino Nista, Sergio Alfieri, Antonio Gasbarrini, Lorenzo Zileri Dal Verme

**Affiliations:** 1 Pancreas Unit, CEMAD Centro Malattie dell’Apparato Digerente, Medicina Interna e Gastroenterologia, Dipartimento di Medicina e Chirurgia Traslazionale, Università Cattolica del Sacro Cuore, Fondazione Policlinico Universitario “A. Gemelli” IRCCS, 00168 Rome, Italymattia.paratore@guest.policlinicogemelli.it (M.P.); marcantonio.negri@policlinicogemelli.it (M.N.); enricocelestino.nista@policlinicogemelli.it (E.C.N.);; 2 Centro Pancreas, Chirurgia Digestiva, Dipartimento di Medicina e Chirurgia Traslazionale, Università Cattolica del Sacro Cuore, Fondazione Policlinico Universitario “A. Gemelli” IRCCS, 00168 Rome, Italysergio.alfieri@unicatt.it (S.A.)

**Keywords:** pancreatic nervous system, pancreatic pain, acute pancreatitis, chronic pancreatitis, pancreatic cancer, neural plasticity, neuromodulation, personalized medicine

## Abstract

The intricate network of the pancreatic nervous system plays a fundamental role in physiologic functions of the endocrine and exocrine pancreas. Several pancreatic diseases affect the normal functionality of the pancreatic nervous system. This chronic derangement leads to anatomical alterations, such as neural hypertrophy and increased nerve density. Perineural invasion is a prominent feature of pancreatic cancer, contributing to cancer progression and metastasis. Despite the fact that these pathogenic mechanisms are still incompletely studied and understood, the constant occurrence of these alterations highlights their importance in the pathophysiology of the pancreatic diseases. The occurrence of anatomical changes is strictly linked to the appearance of pain. Pancreatic pain has peculiar features, and its management is complex in clinical practice. In the present review, the evidence on lifestyle, pharmacological and interventional approaches for the management of pancreatic pain is presented. Analgesic therapy is the cornerstone of pain treatment. However, it is important to identify the individual characteristic of the patients and personalize the approach to pain management. Nevertheless, the incomplete efficacy of these strategies makes this field an area of unmet needs. The study of neuroplasticity is crucial to understand the mechanisms that regulate the pathophysiology of pancreatic diseases. Several trials testing new drugs with specific neuromodulatory effects are ongoing. However, further studies are needed to investigate crucial targets to develop novel therapies for the modulation of the nervous system and the prevention of complications of pancreatic diseases. This comprehensive review summarizes the importance of the nervous system in pancreatic diseases with a special focus on its anatomy and physiology, its pathophysiological features and clinical relevance in pancreatic disease, the treatment of pancreatic pain, and the identification of future trends of research.

## 1. Introduction

Pancreatic diseases are a significant cause of hospitalization, morbidity, and mortality worldwide. The incidence of both benign and malignant pancreatic diseases is increasing in Western countries as a result of the higher prevalence of the risk factors for their development, such as smoking, alcohol intake, physical inactivity, fatty diet, obesity and diabetes mellitus [1,2,3].

The nervous system plays a fundamental role in physiological functions of the endocrine and exocrine pancreas. The complex anatomy of the pancreatic nervous system has been classically classified into two major entities that are strictly interconnected: the intrinsic and extrinsic pancreatic innervation. The intrinsic pancreatic innervation consists of a network of neurons that cluster in the pancreatic ganglia and innervate both the endocrine islets and the exocrine acini of the pancreas with their unmyelinated axons. Parasympathetic, sympathetic, vagal, spinal and entero-pancreatic innervations compose the extrinsic pancreatic nervous system [4,5,6,7].

Several pancreatic diseases affect the normal functionality of the nervous system of the pancreas, resulting in symptoms that are useful indicators of an underlying pancreatic disease. Indeed, the pancreatic nervous system plays a role in both the endocrine and exocrine functions [8]. Although some hypotheses have been made, these pathogenic mechanisms are still incompletely studied and understood. Pain is the most frequent, studied and recognized symptom that is caused by the alteration and/or activation of the pancreatic nervous system [9].

Abdominal pain is a common symptom in both acute and chronic pancreatitis and pancreatic cancer [9]. As for acute pancreatitis, most patients (80–95%) experience an acute onset of severe, persistent epigastric and/or left upper quadrant pain that can be radiated to the right upper quadrant and/or to the back. Prompt recognition of the typical features of pancreatic pain, when associated with a significant increase in serum pancreatic enzymes and/or imaging signs, allows one to diagnose acute pancreatitis [10,11].

Abdominal pain in the early stage of pancreatic cancer is usually milder, vaguer, thus difficult to recognize. Perineural invasion is a prominent feature of pancreatic cancer and often the first route of metastasis. This mechanism is responsible for the occurrence of pain in pancreatic cancer, even at early stages [12]. The incidence of abdominal pain also depends on the stage of the malignancy, occurring in about a third of the patients with early-stage cancer, 60% of patients with locally advanced disease and over 75% of patients with advanced cancer [13,14].

In the diagnostic phase, an expert recognition of the semeiologic features of pain still plays a crucial role in the correct classification of the patient. It is also crucial to identify other guiding features from the anamnestic and physical evaluation and predispose promptly the correct imaging modality with adequate timing. Hence, the understanding of the features of pancreatic pain can be a diagnostic opportunity in the clinical setting, particularly for pancreatic cancer [15].

Importantly, chronic pancreatic pain is also associated with impairments in physical and mental health, affecting the overall quality of life. Indeed, patients with chronic pancreatic pain have a higher prevalence of clinically significant depression, anxiety, sleep disturbance, and physical disability [16].

The management of pain in pancreatic diseases is frequently complex in clinical practice. The absence of large randomized double-blind clinical trials, particularly with the comparison of different drugs, do not allow for a clear indication in international guidelines. Hence, the choice of analgesic strategy is left to clinicians in most cases, and it is often based on their personal experience [17,18]. At present, no pathogenic treatment is available to tackle directly the mechanisms of the pancreatic nervous system impairment in the different pancreatic diseases. For these reasons, this is still a field of unmet needs. Pharmacologic analgesic therapy is the first approach in the vast majority of cases that present with pain. Operative approaches can be indicated in refractory pain in pancreatic cancer and chronic pancreatitis [19,20].

However, pain is just one epiphenomenon of the activation and/or alteration of the nervous system in pancreatic diseases. The exact pathophysiology is still matter of study and debate. Further studies are needed to investigate crucial targets in order to develop new therapies for the modulation of the pancreatic nervous system and the prevention of complications of pancreatic diseases.

The aim of this review article is to summarize the importance of the nervous system in pancreatic diseases, with a special focus on its clinical relevance, the treatment of pancreatic pain, and the identification of future trends of research.

## 2. Anatomy and Physiology of the Pancreatic Nervous System

The pancreas is a richly innervated gland, has a close connection with the central nervous system (CNS) and the enteric nervous system (ENS), and its innervation plays an important role in both exocrine and endocrine physiological function, contributing to maintain homeostasis [21].

The pancreatic neural innervation network is composed of different types of nerve fibers, including spinal and vagal sensory afferent fibers, autonomic fibers divided into a sympathetic and a parasympathetic branch, fibers from the ENS and intrapancreatic ganglia. Spinal and vagal sensory nerves process metabolic signals and send sensory inputs to the CNS, which exerts an important function of coordination and the integration of stimuli via the ascending nerves and subsequently controls internal organs and glands via descending nerves. On the contrary, sympathetic and parasympathetic branches of the autonomic nervous system have an important role in regulating reflexive and involuntary organ functions [21].

### 2.1. Spinal and Vagal Sensory Afferent Fibers

Both spinal and vagal pathways are responsible for releasing the tissue information to CNS and regulating pancreatic functions and homeostasis through neuropeptides. Spinal and vagal sensory afferent fibers consist of small-diameter Ad or C fibers that respectively originate from the pseudounipolar neurons in the dorsal root ganglia (DRG) and thoracic 6 (T6)—lumbar 2 (L2) segmental levels of the spinal cord and the pseudounipolar sensory neurons in the bilateral nodose ganglia. Sympathetic afferent fibers of the spinal nerve subsequently create synapses in lamina I and IV in the dorsal horn at the same spinal segment and project to neurons in the intermediolateral column of the spinal cord, whereas sympathetic afferent fibers of the vagus project from blood vessels, ducts, acini, islets, and intrapancreatic ganglia without building neural synapses [22,23].

The sympathetic afferent fibers express the transient receptor potential cation channel vanilloid 1 (TRPV1) and produce substance P, calcitonin gene-related peptide (CGRP) and neuropeptide Y (NPY) which are involved in sensing both chemical and mechanical signals, control metabolic homeostasis and pain, and regulate inflammatory processes [24,25,26]. The influence of vagal stimulation on endocrine secretion is still debated [27].

### 2.2. Sympathetic and Parasympathetic Autonomic Fibers

The autonomic pancreatic innervation is divided into two different branches including both sympathetic and parasympathetic fibers [6]. The cell bodies of the lower thoracic and upper lumbar segments of the spinal cord represent the origin of preganglionic efferent sympathetic fibers that run along the splanchnic nerves, releasing acetylcholine and forming synapses within the prevertebral ganglia [28,29]. From celiac and superior mesenteric ganglia, the post-ganglionic sympathetic fibers enter directly the pancreas, projecting to intrapancreatic ganglia, islets, ducts, blood vessels, and exocrine pancreas to a lesser extent. They release several neurotransmitters, such as norepinephrine, galanin, and neuropeptide Y. They are involved in the development and functional maturation of pancreatic islets [30]. The adrenergic sympathetic stimulation seems to be involved in the inhibition of pancreatic exocrine secretion through vasoconstriction effects on the capillary beds and predominantly in the regulation of pancreatic β-cell function and glucose homeostasis [31].

The left medial and lateral cell column of the dorsal motor nucleus of the vagus represent the main origin of the efferent parasympathetic fibers, but they can also originate in the nucleus ambiguous. They travel mostly in hepatic and bilateral gastric branches, and relatively few travel in the bilateral celiac branches of the vagus and terminate in the intrapancreatic ganglia or enter the islet from the parenchyma, creating synapses with nicotinic receptors. Postganglionic parasympathetic fibers from the intrapancreatic ganglia reach islets, acini, and vasculature expressing muscarinic type 1 and 3 acetylcholine receptors and release acetylcholine that stimulates glucagon-secreting a-cells, insulin-secreting b-cells, somatostatin-secreting d-cells, exocrine acinar cells, and epithelia of the duct system [32,33]. They also release vasoactive intestinal polypeptide, gastrin-releasing peptide, pituitary adenylate cyclase-activating polypeptide that are able to stimulate and increase the secretion of glucagon and insulin and neuropeptide Y, galanin, and nitric oxide (NO), which negatively influence pancreatic endocrine and exocrine secretion [34].

### 2.3. Fibers from the Enteric Nervous System (ENS)

The ENS is located within the wall of the gastrointestinal tract. It is composed of the myenteric plexus and the submucous plexus, which act as mechanosensors and chemosensors [35]. It is considered the “local brain” that regulates several gastrointestinal functions, strengthening pancreatic secretion through the effects of neuromodulators, such as pituitary adenylate cyclase-activating peptides, or inhibiting amylase secretion by axo-axonic synapses formed by the fibers of serotoninergic enteropancreatic neurons. Some of the enteropancreatic neurons are cholinergic and form excitatory nicotinic synapses on neurons in intrapancreatic ganglia [36,37].

### 2.4. Intrapancreatic Ganglia

Intrapancreatic ganglia are composed of 1–35 nerve cells that are located near nerve trunks in the interlobular, acinar and within lobules and islets of the pancreatic parenchyma, predominantly in the perivascular plexus and less in the perineural plexus. The intrapancreatic neurons receive inputs from the parasympathetic, sympathetic, sensory and enteric neurons. Their fibers reach vasculatures, endocrine cells in islets, exocrine acini and the ductal system [38,39]. They release acetylcholine to promote exocrine secretion from acinar cells and endocrine secretion of insulin and glucagon from endocrine cells. They also synthesize a large number of biologically active substances including neuropeptide Y, CGRP, NO, vasoactive intestinal polypeptide, norepinephrine, enkephalin, and gastrin-releasing peptide which mediate different physiological responses [40].

## 3. Neuroplasticity and Neural Invasion

The ability of the nervous system to adapt and reshape in response to acute stimuli is called “neuroplasticity”. The term “neural plasticity” encompasses morphological and/or functional changes in nerves (morphology, density and/or quality of fibers) in response to *noxae*. Neuronal plasticity refers to alterations at the cellular level [41].

**Figure 1:**
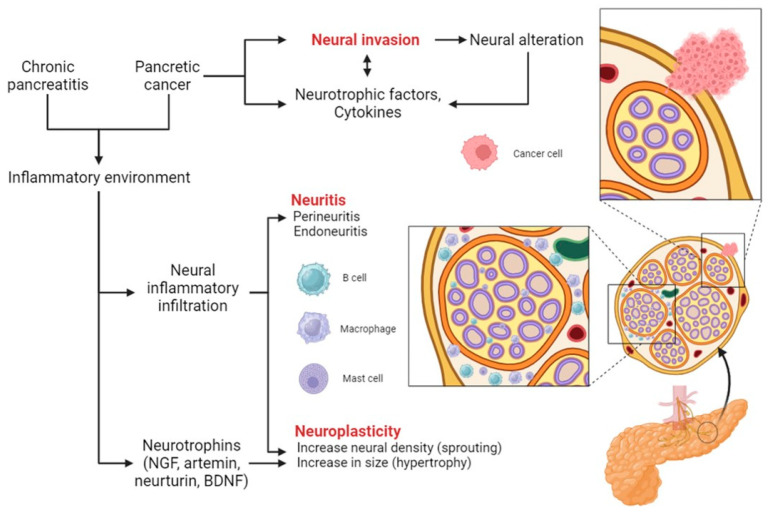
Mechanisms of neuroplasticity and neural invasion in pancreatic diseases. Abbreviations: NGF, neural growth factor; BDNF, brain-derived neurotrophic factor. Created with BioRender.com.

In chronic pancreatitis and pancreatic cancer, an increase in neural density (neural sprouting) and size (neural hypertrophy) are typically observed compared with healthy individuals [42,43]. Indeed, the inflammatory environment of pancreatic cancer and chronic pancreatitis stimulates the production of neurotrophic mediators, such as nerve growth factor (NGF), artemin, and neurturin. The upregulation of neurotrophic factors, such as brain-derived neurotrophic factor (BDNF) and artemin, has been associated with the severity of perineural inflammatory cell infiltration, increased neural density and hypertrophy, accelerating the formation of enlarged intrapancreatic nerves and decreasing the threshold for the transmission of pain [44]. Moreover, the extent of nerve alterations correlates with the expression of these neurotrophins [45,46] (Figure 1).

In addition, neural infiltration by inflammatory cells is common in both pancreatic cancer and chronic pancreatitis [46]. It can be distinguished into the following: perineuritis, when the immune cells are localized around the perineurium, and endoneuritis, when they can be observed among the nerve fibers. CD8^+^ cytotoxic T cells, macrophages and mast cells are the most prevalent infiltrates. In particular, the presence of mast cells correlates with pain [47]. Interestingly, they can establish a specific mutual crosstalk with nerve terminals, which is mediated by histamine, 5-hydroxytryptamine, and cytokines from the one side and neuropeptide from the other [48,49]. Importantly, mast cells are also involved in the pathogenesis of neuropathic pain in other gastroenterological diseases [50].

The entity of pancreatic neuritis correlates with neural density and pain [46]. In this process, intracellular IL-8 is increased in immune cells, and substance P is more abundant in infiltrated nerves. Besides being a pain mediator, substance P seems to be implicated in the development of neurogenic inflammation, a mechanism that can perpetuate pain [51].

Several studies investigating cortical activity revealed important features of central neuroplasticity [41]. In particular, patients with chronic pancreatitis showed central hyperalgesia (remote hyperalgesia to electric or heat stimulation) [52], impaired descending inhibitory pain and diffuse noxious inhibitory control [52], an altered brain resting activity and rhythmicity on electroencephalograms (increased activity in the lower frequency bands δ and lower peak α frequency) [53,54], altered contact heat-evoked potentials with prolonged latencies [55,56,57], an altered brain microstructure as assessed via a diffusion tensor MRI in brain regions such as the amygdala, cingulate cortex, insula, prefrontal and secondary sensory cortex [58], and an altered brain macrostructure (reduced cortical thickness in the secondary somatosensory cortex, prefrontal cortex, frontal cortex, mid and posterior cingulated cortex regions as assessed via MRI) [59].

Little is still known about the influence of glial cells in the development of neuroplasticity [41].

The interaction between cancer cells and nerves by means of neurotrophic factor and cytokines may promote mutual growth and proliferation. In patients with gastric and prostatic cancer, nerve density correlated with tumor stage and serum biomarkers. Vagotomy and anticholinergic therapy were associated with an improved survival in preclinical models [60,61]. Hence, it has been hypothesized that these treatments may be effective in the clinical setting [41].

Neural invasion is a prominent feature of pancreatic cancer, which occurs in almost all cases. It is an early event in carcinogenesis: in fact, it is already present in 75% of stage I pancreatic cancer [62]. The direct infiltration of nerves by cancer cells is the primary mechanism for the development of neural alterations and pain in pancreatic cancer. In addition, cancer cells produce a variety of neurotrophic factors that further favor the irritation and consequent activation of neural terminals [43,63] (Figure 1).

Pancreatic cancer cells proliferate and metastasize not only by following peripherally the route of the nerves but also penetrating the perineurium and endoneurium. Several neurotrophic factors, chemokines and cell-surface ligands and receptors have been investigated in their potential role in neuro-cancer interactions. The increased expression of transforming growth factor-α (TGF-α) in the nerves surrounding the pancreas and epidermal growth factor receptor (EGFR) in pancreatic cancer cells were shown to increase the affinity of nerves for pancreatic cancer cells and vice versa [64].

NGF and tirosinkinase A (TrkA) mRNA levels are markedly overexpressed in patients with perineural invasion and with a higher degree of pain. Hence, neurotrophic factors may increase the invasiveness and proliferation of cancer cells, highlighting the mutual crosstalk between cancer cells and nerves, increasing the invasiveness of pancreatic cancer cells [65,66].

The glial cell line-derived neurotrophic factor (GDNF) family of growth factors is also involved in neural invasion. GDNF is linked to a ligand-bound GFRα receptor and the rearranged during transfection (RET) receptor tyrosine kinase, which was originally discovered as a proto-oncogene, activating signaling pathways that control cell growth, differentiation, and neurite outgrowth and survival and increasing the invasiveness of pancreatic cancer cells towards pancreatic nerves [67,68].

Also, the chemokine receptor CX3CR1 and its ligand CX3CL1 were associated with an increased incidence of neural invasion in pancreatic cancer [69].

## 4. Pathophysiology of Pancreatic Pain

Different mechanisms can cause pain in pancreatic diseases, resulting in different types of pain: nociceptive pain, neuropathic pain, and neurogenic inflammation.

Nociceptive pain occurs as a result of the stimulation of primary afferent neurons in response to a chemical or mechanical activation. The resulting pain is proportional to stimulation. Inflammation, ischemia, and increased pressure are the main causes of this kind of pain. Bradykinins, prostaglandins and substance P are the most frequent mediators of nociceptive pain. The obstruction of pancreatic or biliary ducts and the consequent increase in ductal pressure can also cause nociceptive pain [70]. In early studies, this mechanism (“plumbing” theory) was considered particularly important in the pathophysiology of pain in both benign and malignant pancreatic diseases. However, (1) significant dilation (>5 mm) is present in about 30% of cases of chronic calcifying pancreatitis [71], (2) similar duct dilation results can be observed both in painless and painful chronic pancreatitis [72,73], and (3) decompression is associated with improved pain in only 50% of cases [70,74].

The development of pancreatic neuropathy, which is characterized by changes in nerve morphology and the disturbance of neural homeostasis, is typical of both chronic pancreatitis and pancreatic cancers, suggesting that pain in these diseases may have a neuropathic component [41]. Neuropathic pain is the consequence of an alteration of sensitive nerves that usually happens in response to chronic nociceptive activation. This mechanism is responsible for hyperalgesia in prolonged pain stimulation. Neuropathic pain has a complex pathogenesis that is not fully elucidated. Several biochemical mediators, particularly ATP, colony-stimulating factor 1, and brain-derived neurotrophic factor, seem to have a role in the initiation. The perpetuation of the biochemical and structural nerve alterations causes the peripheral sensitization [75].

In neurogenic inflammation, biochemical changes in the nerve microenvironment produce the release of cytokines and neuropeptides from the nerve, enhancing inflammation and consequent pain [76,77]. It seems to be mediated by the effect of the neuropeptide substance P that is released by sensory C fibers into the spinal cord and acts through its specific G protein-coupled neurokinin receptors, promoting central hyperexcitability (central sensitization), increasing pain perception (hyperalgesia and allodynia) and contributing to the damage of the perineurium and the infiltration of the nerves by immune cells [51,78]. Higher immunoreactivity for substance P and increased levels of neurokinin receptors mRNA in patients with chronic pancreatitis have been demonstrated to have a strong correlation with the intensity, frequency and duration of pain [79,80]. Moreover, the expression of NGF and its high-affinity receptor TrkA is markedly increased in nerves, ganglia and in the perineurium of intrapancreatic nerves, with a significant correlation with their mRNA levels, pancreatic fibrosis and acinar cell damage [81].

Anatomically, regardless of the precise mechanism that generates the dolorific stimulus, it travels through single nerve fibers that cluster together with fibers coming from other splanchnic organs, forming the primary sensory nerves that reach the spinal cord [82]. The clustering of nerves determines a significant overlap in signals from different organs, making the localization of the pain vague. Hence, the correct interpretation of the originating organ can be hard in some cases [83].

The mechanisms of neuropathic pain and neurogenic inflammation determine the peripheral sensitization. Afterwards, the pathogenic mechanism is enhanced and propagated in the spinal dorsal horn and throughout the somatosensory system, causing the central sensitization that maintains the process in the long term.

**Figure 2:**
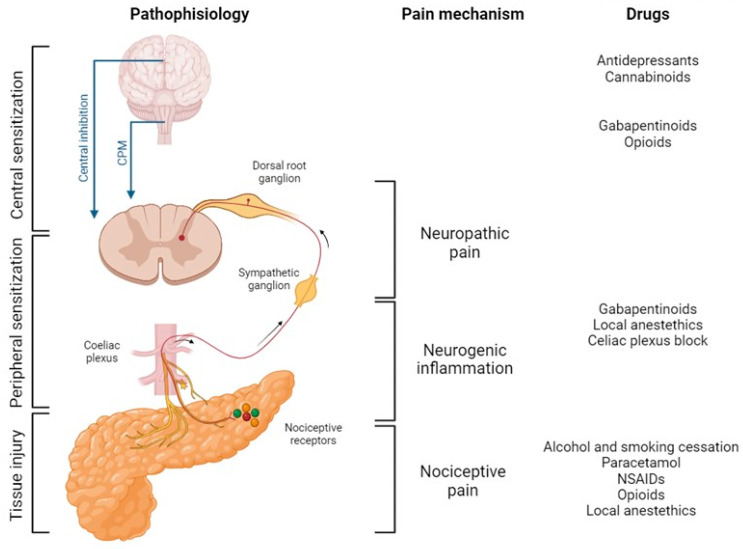
Pathophysiology, mechanisms, and drugs for pancreatic pain. This figure was inspired by and based on the figures in references [87,88]. Abbreviations: CPM, conditioned pain modulation; NSAIDs, non-steroidal anti-inflammatory drugs. Created with BioRender.com.

Abnormal pain processing and central neuroplastic changes because of the repeated and pathological activation of the afferent pathways increase the activity in several pain-related nervous system areas. Hence, when central sensitization occurs, these areas remain active regardless of the real peripheral nerve activation [84]. This evidence is supported by the fact that total pancreatectomy fails to alleviate the pain in up to 30% of CP patients and that permanent ablation of primary sensory neurons by capsaicin alleviates inflammation [85,86] (Figure 2).

## 5. Lifestyle, Pharmacological, and Interventional Management of Pain in Pancreatic Diseases

Pain is an extremely common symptom in pancreatic diseases. However, precise indications regarding pain management are lacking in the main international guidelines on acute pancreatitis [18] and pancreatic cancer [89]. Hence, the ladder approach of the WHO to pain management is generally applied [90]. Expert consultations by pain management units are generally recommended when pain remains uncontrolled despite first-line strategies. As for acute pancreatitis, this approach was also associated with a shorter hospital stay [91]. Conversely, specific guidelines were developed for pain management in chronic pancreatitis [20].

### 5.1. Treatment of Complications

The occurrence of complications in both acute (fluid collections, pancreatic necrosis, infected necrosis, biliary tract obstruction and/or infections) and chronic pancreatitis (pseudocysts, ductal pancreatic stones, biliary obstruction, gastric outlet obstruction) and pancreatic cancer (biliary tract obstruction and/or infections, gastric outlet obstruction, duodenal infiltration and occlusion) is frequent. Complications are very frequently associated with pain. Hence, they often require a specific treatment in order to achieve pain relief [20,89,92] (Table 1).

### 5.2. Lifestyle Interventions

#### 5.2.1. Diet

Despite the common perpetuation of the traditional prolonged fasting approach during the early days in clinical practice, international guidelines recommend to start oral feeding again as early as possible in predicted mild and moderate acute pancreatitis, regardless of pain resolution or lipase levels. The initial diet should contain a low amount of fat [93]. The delay of refeeding is associated with a higher risk of complications and a longer hospital stay [94]. Moreover, a recent randomized controlled trial showed a significant benefit with the immediate resumption of a solid diet [95]. In a meta-analysis, 16.3% of patients with acute pancreatitis developed food intolerance. This condition was associated with higher severity and/or clinical complications [96].

In case of oral diet intolerance, enteral nutrition via a nasogastric tube should be administered. Parenteral nutrition should be limited to patients with intolerance or contraindications to enteral nutrition [93].

Historically, a low-fat diet was recommended in patients with chronic pancreatitis. However, a standard fatty diet is well tolerated and should be recommended in all patients [93,97]. Frequently, inadequate pain control is associated with a suboptimal dietary intake and malnutrition [98]. Nutritional intervention can improve nutritional biomarkers and reduce pain [97].

As for pancreatic cancer, no restrictive diet should be prescribed. Oral nutritional supplements are useful tools to contrast cachexia and sarcopenia [99].

#### 5.2.2. Alcohol and Smoking Cessation

High alcohol consumption and smoking are risk factors for the development of both acute and chronic pancreatitis [100,101,102], and the cessation of alcohol intake is associated with a reduction in recurrences [103]. Smoking is an independent risk factor for pancreatitis, and it also potentiates the toxic effects of alcohol on the pancreas in a dose-dependent fashion [101,104]. Moreover, it is an independent risk factor for the development of pancreatic cancer. However, while smoking cessation has a strong rationale and is largely recommended by experts, the effect of smoking cessation on pain has not been proven in clinical trials [20].

Both alcohol and smoking cessation can be obtained via pharmacological and non-pharmacological approaches. Benzodiazepines and mood stabilizers are used for the treatment of alcohol withdrawal symptoms. Naltrexone is the most evidence-supported and widely used alcohol anti-craving therapy. Nicotine receptor agonists are the most effective pharmacological treatments for smoking cessation. Cognitive behavioral therapies are recommended as non-pharmacological therapies for the achievement of both alcohol and smoking cessation [20].

### 5.3. Pharmacological Strategies

Pharmacological classes and drugs for the management of pancreatic pain are reported in Table 2.

#### 5.3.1. Pancreatic Enzyme Replacement Therapy (PERT)

Digestive intolerance due to pancreatic exocrine insufficiency (PEI), which frequently manifests with bloating and abdominal pain, should be recognized promptly and treated with pancreatic enzyme replacement therapy (PERT). The incidence of PEI is very common in patients with advanced chronic pancreatitis and pancreatic cancer, particularly when it is located in the head of the gland. It can be difficult to distinguish nociceptive or neuropathic pain from pain related to PEI. Hence, it is important to always consider the possibility of PEI, particularly in patients with a high probability of PEI [93,98]. Patients with pancreatic cancer with PEI benefit from adequate PERT in terms of reduced pain, higher quality of life, and longer survival [105].

#### 5.3.2. Paracetamol

Paracetamol has limited efficacy on pancreatic pain management, particularly in the setting of acute pancreatitis. It exerts no activity on cyclooxygenase (COX) and thus has no anti-inflammatory effect. However, paracetamol has a broad background effect, limited contraindications and side effects, and dose-dependent toxicity. It is the first step in the step-up-approach ladder for pain management by the World Health Organization (WHO). Hence, it is frequently used as an adjuvant therapy in association with other analgesics in pancreatic diseases [20,106].

#### 5.3.3. Non-Steroidal Anti-Inflammatory Drugs (NSAIDs)

Non-steroidal anti-inflammatory drugs (NSAIDs) are reversible cyclooxygenase (COX) 1 and 2 inhibitors, thus reducing the production of prostaglandins and leukotrienes that act as inflammatory mediators. Hence, they possess anti-inflammatory, analgesic, and antipyretic activity. They act on nociceptive pain, which is frequently caused by inflammation, the main pathogenic driver in acute and chronic pancreatitis. However, NSAIDs are burdened with significant side effects, such as gastrointestinal ulcers, acute kidney injury, and hypertensive emergencies. Hence, their use should be carefully evaluated in the setting of patients with pancreatic diseases, who are at high risk of severe complications [107]. Indeed, the 2019 guidelines for the management of severe acute pancreatitis of the World Society of Emergency Surgery (WSES) recommends against the use of NSAIDs in patients with acute kidney injury [108]. In two recent meta-analyses, NSAIDs and opioids were equally effective in achieving pain relief in acute pancreatitis. No significant difference was observed in the need for rescue analgesia with either of these analgesic agents [18,106].

#### 5.3.4. Opioids

Opioids exert an agonist activity on three main classes of receptors (mu, delta, and kappa) that are expressed on both central and peripheral neurons, neuroendocrine and immune cells. Opioid receptor binding activates pre- and post-synaptic Ca^2+^ channels, reducing the neuronal excitability and the production of pronociceptive neuropeptides. Interestingly, opioid receptor expression on peripheral nerves (both in terms of number per neuron and number of neurons expressing receptors) increases in response to long-term tissue inflammation. The effect of opioids has been studied in vitro, but in vivo studies are still inconclusive and debatable [109,110]. The study of neuronal receptor plasticity has a significant potential for the understanding of nerve injury and neuroinflammation mechanisms and their modulation.

Traditionally, opioids were considered contraindicated in pancreatic diseases due to the initial evidence that they caused spasms of the sphincter of Oddi and the consequent risk of perpetuating pancreatic duct obstruction. However, studies using manometry during endoscopic retrograde cholangio-pancreatography (ERCP) deflated the results of older studies based on radiographic and contrastographic techniques [111,112].

Nowadays, opioids are a cornerstone of analgesic therapy for acute pancreatitis with severe pain. Different opioids have been tested in trials with some controversial results and no clear evidence of superiority of one agent over the others. However, most of these trials were burdened by small numbers and heterogeneity of the severity of the pancreatitis [18,106]. Tramadol, buprenorphine, oxycodone, and fentanyl are the most widely used drugs in clinical practice. Recently, a clinical trial showed that buprenorphine was superior to diclofenac in controlling pain in acute pancreatitis (outcome of fentanyl rescue therapy: 32 [IQR 21–69] vs. 8 [IQR 4–15], *p* < 0.001) with a similar safety profile [113].

In a recent meta-analysis, the use of opioids reduced the need for rescue analgesia compared with non-opioids in patients with acute pancreatitis. However, heterogeneous pain therapies with different predicted efficacy, such as paracetamol, local anesthetics, and NSAIDs, were grouped in the non-opioids class. As mentioned above, both opioids and NSAIDs were equally effective in this setting [18,106].

In the management of chronic pain, the use of opioids should be carefully evaluated due to common gastrointestinal side effects and the risk for addiction in patients with a significant predisposition to depression and mood disorders [114,115,116].

Tramadol is the most widely preferred second-line therapy for pain management in chronic pancreatitis. It owns a weak opioid agonist activity and has weak effects on noradrenalin and serotonin reuptake, thus tackling different mechanisms. Its efficacy in controlling pain was rated higher by patients compared with morphine for the same activity of analgesia and had less impact on gastrointestinal motility [117,118]. However, only about 25% of patients with chronic pancreatitis benefit from long-term opioid therapy. Moreover, it must be considered that a significant interindividual variability in responsiveness to different opioids and tolerance/habituation is common, requiring occasional drug rotations and dose titrations [20].

The intensity and chronicity of cancer pain often requires the step up to strong opioids. Particularly, strong agonist activity opioids are frequently prescribed at diagnosis for moderate to severe pain [119]. In a recent study of a large cohort of patients, the use of opioids was independently associated with longer survival (6.0 vs. 4.0 months; *p* < 0.0001) [120].

It is debated whether opioids can induce hyperalgesia (opioid-induced hyperalgesia, OIH) after long treatments. It consists of an increased predisposition to pain and enhanced pain perception. Recent evidence suggests that it may be rather caused by withdrawal-induced hyperalgesia after abrupt cessations of opioids. This often leads to increasing the dose of opioids and extending the course of the treatment. Anecdotal cases of allodynia have been attributed to the neuroexcitatory effects of some metabolites at extremely high doses of opioids [121,122,123].

#### 5.3.5. Gabapentinoids

In the clinical practice setting, gabapentinoids are retained as first-line treatments for neuropathic pain. Their mechanism of action is not fully understood. However, they probably exert effects on multiple targets of neurons, particularly ion channels [75]. Hence, gabapentinoids are frequently prescribed in different clinical conditions when a neuropathic component is suspected.

In a randomized controlled trial, the use of pregabalin (150 mg b.i.d., which is then titrated up to 300 mg b.i.d.) showed better pain control (36% vs. 24% measured via a visual analog scale; *p* = 0.02) in patients with chronic pancreatitis compared with the placebo. Similarly, the health status score Patients’ Global Impression of Change (PGIC) was improved (44% vs. 21%; *p* = 0.048) [124]. Hence, it can be considered an adjuvant therapy for pain management. There is some evidence on the use of gabapentin in rat models of chronic pancreatitis [125], but it has not been tested in clinical trials yet.

#### 5.3.6. Local Anesthetics

The use of systemically administered local anesthetics was only tested in patients with acute pancreatitis.

In a randomized controlled trial, the systemic administration of procaine, a local anesthetic, was associated with better pain control compared with the placebo (visual analogue scale (VAS) decrease of −62 vs. −39; *p* = 0.025). This approach was also associated with a subjective achievement of pain control (*p* = 0.018) reduction in the use of other analgesics (*p* = 0.042) and a lower risk of hospitalization (−80%, *p* = 0.012) [126]. However, another randomized controlled trial showed no significant benefit compared with the placebo in terms of rescue analgesia demand (buprenorphine) [127]. Procaine was also less efficient than buprenorphine in achieving pain control in terms of the need for rescue analgesia (*p* < 0.0001), pain score reduction (*p* < 0.05), and rapidity of the analgesic effect (*p* < 0.0001) [128]. Similarly, patients in the procaine arm experienced higher pain scores in the first 3 days of treatment compared with patients in the pentazocine arm (*p* < 0.001) in another trial [129].

Based on these results, systemically administered local anesthetics are not routinely recommended for the management of pancreatic pain.

#### 5.3.7. Antidepressants

When central sensitization and a consequent altered processing of chronic pancreatic pain occurs, a specific treatment with antidepressants is generally indicated. Tricyclic antidepressants are the most commonly prescribed drugs [87,88]. Duloxetin, an erotonin-noradrenalin reuptake inhibitor, is another option for the treatment of depression in pancreatic diseases [130]. However, there are no controlled trials on their use in pancreatic diseases.

#### 5.3.8. Cannabinoids

The cannabinoids dronabinol and nabilone are approved for the treatment of cancer-related symptoms [131]. They exert their effect through the binding of cannabinoid receptors 1 (CB1), which are located in brain regions controlling nociceptive processing and peripheral nerve terminals, and 2 (CB2), which reduce levels of inflammatory cytokines and increase the release of endogenous opioids [132]. Besides improving pain control, they combat anorexia and favor sleep [133]. They can be considered as an adjuvant therapy for selected patients with pancreatic cancer after consultation with a pain management expert.

### 5.4. Interventional Approaches

#### 5.4.1. Epidural Analgesia

Epidural analgesia (bupivacaine 0.1% + fentanyl 2 µg/h with a continuous infusion of 6–15 mL/h and 3–5 mL bolus on demand) was more effective than patient-controlled intravenous analgesia in controlling pain in acute pancreatitis. This result was statistically significant after the epidural analgesia implementation (VAS 1.6 vs. 3.5; *p* = 0.02) and at 10 days (0.2 vs. 2.33; *p* = 0.034). No complications with the epidural procedure were observed [134].

In a recent meta-analysis, epidural analgesia was the most effective pain treatment for acute pancreatitis during the first 24 hours in acute pancreatitis [106].

In the intensive care setting, the use of epidural analgesia was an independent predictor of reduced mortality at 30 days (adjusted odds ratio 0.10; [95% CI, 0.02–0.49]; *p* = 0.004), suggesting a possible therapeutic effect [135]. In another trial, it was not associated with a reduction in the need for ventilation compared with controls [136].

#### 5.4.2. Celiac Plexus Block (CPB)

Celiac plexus block (CPB) or neurolysis is an interventional technique to block celiac plexus nerves via mechanical or chemical destruction. It can be achieved by either CT-guided percutaneous procedures, endoscopic ultrasonography (EUS), or surgical (often referred to as splanchnicectomy) approaches. CPB is a palliative procedure that is usually considered when the medical management fails to achieve pain relief. Bupivacaine 0.25% 10–20 mL followed by 10–20 mL 98% ethanol is the most widely used neurolytic protocol [19].

In a recent meta-analysis, CPB was superior to standard analgesic therapy in the outcome of VAS at 4 weeks (−0.43, 95% CI −0.73–−0.14, *p* = 0.004), VAS at 8 weeks (−0.44; [95% CI—−0.89–0.01]; *p* = 0.06), opioid use at 8 weeks (−51.07%; [95% CI −82.71–−19.43]; *p* = 0.002), and opioid use the day before the death (−48.52; [95% CI −68.82–−28.22]; *p* < 0.00001). Although the opioids were not discontinued, their dosage was significantly reduced as well as the occurrence of their side effects (*p* < 0.00001) [137]. Another meta-analysis demonstrated a similar reduction in the use of analgesics, but it was statistically significant only at 4 weeks after the CPB [138].

The efficacy of EUS-CPB in achieving pain relief ranges from 50 to 94%. However, it is frequently limited over time [19].

As for chronic pancreatitis, CPB is rarely used in clinical practice. Indeed, the limited efficacy in the long term, procedural risks (e.g., bleeding, infections) and side effects (e.g., postural hypotension, diarrhea) make it more suitable for the palliative care of patients with malignancies [19,20]. One trial compared the EUS- and CT-guided approaches in patients with chronic pancreatitis. Patients experienced a higher rate of analgesics reduction with the EUS-guided compared with CT-guided CPB (50 vs. 25%). It was also the preferred approach of patients who underwent both techniques. Finally, the safety was comparable [139].

#### 5.4.3. Spinal Cord Stimulation

During the last 20 years, several successful treatments of chronic pancreatitis pain with spinal cord stimulation were reported. A recent meta-analysis synthetized the evidence on the use of this technique. All patients experienced a decrease in pain with a median VAS reduction of 61% and a morphine-equivalent use of 69%. The main adverse events were implant site infections (6%) and lead migration (6%) [140].

Nevertheless, this approach should be considered in a personalized approach in highly experienced centers.

## 6. Future Trends in Neuropancreatology: Pharmacological Neuromodulation

The transition from symptomatic therapies to pathogenic treatments is always crucial for the achievement of better clinical results. However, this step requires strong preclinical studies to understand the pathogenesis, the identification of potential treatments, and trials with adequate outcomes to verify the clinical benefit.

Several mitogen-activated protein kinases (MAPKs), a family of intracellular signaling pathways, are involved in the transduction of signals in neuropathic pain pathways. In particular, p38 MAPK, extracellular signal-regulated kinases 1 and 2 (ERK1/2), and c-Jun N-terminal kinase (JNK) have been proposed as potential targets for therapy. Moreover, nuclear factor kappa B (NF-κB), nuclear factor erythroid 2-related factor 2 (NRF2), and phosphoinositide 3-kinase (PI3K) seem to play a significant role in other relevant pathways. Based on this evidence, minocycline, a MAPK and matrix metalloproteinase (MMP) modulator, astaxanthin, a MAPK and NRF2 modulator, peimine, a MAPK and NF-κB modulator, and fisetin, a MAPK, NF-κB and PI3K modulator, have been tested as neuromodulators in preclinical models with interesting results. They need to be tested in clinical trials to assess their safety and efficacy in the clinical setting [141].

Neuromodulation may also be an option for treating inflammation and pain in pancreatitis. Indeed, the activation of α7 nicotinic acetylcholine receptors can reduce pain and inflammation in experimental models of pancreatitis [142,143]. Hence, it would be interesting to test this strategy in clinical trials [144].

Considering the importance of neural invasion in the progression of pancreatic cancer and its clinical implications in terms of symptoms, it is an attractive target for treatment interventions. In this context, the NGF-TrkA pathway is the most studied for therapeutic approaches [145]. Based on preclinical studies [146], the monoclonal antibody tanezumab targeting NGF is currently studied in controlling pain in patients with bone metastases (e.g., ClinicalTrials.gov NCT00545129). Similarly, the monoclonal antibody MNAC13, which has high affinity with the TrkA receptor, in association with opioids, was associated with pain relief in a murine model [147]. In addition, ARRY-470, a pan-tyrosinkinase inhibitor, was able to reduce pain and nerve remodeling in a murine model of sarcoma [148].

TrkA inhibitors may also act as direct anticancer agents. PHA-848125, a dual inhibitor of cyclin-dependent kinases (CDKs) and TrkA, is currently being evaluated in phase I and II trials after positive results in inhibiting cancer growth in xenograft models. It has also been associated with better pain control in pancreatic cancer [149].

The TRPV1 pathway is another possible target to be tackled pharmacologically. Resiniferatoxin, a capsaicin analogue antagonizing TRPV1, can induce apoptosis and reduce pain in pancreatic cancer [150]. Considering the importance of TRPV1 in the pathogenesis of neurogenic inflammation, these drugs may be also helpful in the neuromodulation and management of pain in pancreatitis [76].

Several studies demonstrated a carcinogenic effect of sympathetic nervous system activation in pancreatic cancer. In particular, β-adrenergic receptors stimulate pancreatic cancer cells proliferation and migration. They are also able to upregulate the α7 nicotinic acetylcholine receptor. Hence, unselective β-blockers may be a promising adjuvant oncologic therapy to be tested in clinical trials [151,152].

Epigenetic regulation may also plays a role in neuromodulation. Inhibitors of histone deacetylases (HDACs) were recently studied as potential anticancer drugs. In animal models, they also showed anti-inflammatory and neuromodulatory effects. Neuropathic pain is often caused by a derangement in the GABA-mediated inhibitory effect, which is also associated with reduced acetylation. Hence, HDAC inhibitors may restore GABA-mediated antinociception, favoring pain relief [153,154].

## 7. Conclusions

The pancreatic nervous system plays a crucial role in the pancreatic physiology. Pancreatic diseases derange the normal physiology of the nervous system in the pancreas, resulting in pain and impairment of the endocrine and exocrine pancreatic functions.

The study of the pathophysiology of the pancreatic nervous system may help to identify targets for the development of specific treatments. For this purpose, research consortia of international pancreatic societies have been constituted to study pancreatic pain [155]. Future trends of research may reveal mechanisms of regulation that may be modulated with specific therapies or highlight pathophysiologic derangements to be tackled directly. Hence, translational research is crucial in this field in order to develop treatments to improve the quality of life of patients with pancreatic diseases.

## Figures and Tables

**Table 1:** Complications of pancreatic diseases and their treatments.

Complication	Indication for Treatment	First-Line Treatment	Alternative Therapies
Pseudocyst Fluid collection Walled-off necrosis	Symptoms (e.g., pain, gastric outlet obstruction, intestinal obstruction) Vascular obstruction Infection	EUS-guided stent positioning	US/CT-guided percutaneous drainage Surgery
Main biliary duct obstruction	Jaundice Increased cholestatic LFTs Infection	ERCP	EUS-guided stent positioning US-guided percutaneous biliary drainage
Pancreatic ductal stones	Pain, evidence of duct dilation, and inadequate pain control	ESWL	ESWL + ERCP
Pancreatic ductal stricture	Pain, evidence of duct dilation, and inadequate pain control	ERCP	Surgery
Gastric outlet obstruction	Symptoms (vomiting, pain)	Surgical gastroenterostomy Endoscopic stenting (pancreatic cancer)	EUS-guided gastroenterostomy Surgical gastroenterostomy

Abbreviations: ERCP, endoscopic retrograde cholangio-pancreatography; ESWL, extracorporeal shock-wave lithotripsy; EUS, endoscopic ultrasonography; LFTs, liver function tests; US, ultrasonography.

**Table 2:** Pharmacological classes and drugs for the management of pancreatic pain.

Pharmacological Class	Drug and Dosage	Possible Side Effects	Disease
Antipyretics	Paracetamol 1000 mg (usually t.i.d. to obtain analgesic effect)	Thrombocytopenia, leukopenia, anemia, acute hepatitis, anaphylactic shock, hypotension, dizziness	Acute pancreatitis Chronic pancreatitis Pancreatic cancer
NSAIDs	Ibuprofen 600 mg qd. (up to t.i.d.) Ketoprofen 50 mg qd. (up to q.i.d.) Ketorolac 10 mg qd. (up to q.i.d.)	Peptic ulcer, gastrointestinal bleeding, vomiting, nausea, diarrhea, dyspepsia, skin rash, fatigue, acute kidney injury, hypertension	Acute pancreatitis Chronic pancreatitis Pancreatic cancer
Opioids	Tramadol 50 mg qd. (up to q.i.d.) Oxycodone 10 mg (up to 80 mg) b.i.d. Fentanyl 100 mcg sublingual route (up to 400 mcg) q.i.d. Fentanyl transdermal route 25 (up to 100) mcg/h	Drowsiness, headache, dizziness, constipation, nausea, fatigue, dry mouth, vomiting, hyperhidrosis	Acute pancreatitis Chronic pancreatitis Pancreatic cancer
Gabapentinoids	Pregabalin 150 mg (up to 300 mg) b.i.d.	Viral infections, leukopenia, anorexia, increased appetite, confusion, depression, anxiety, drowsiness, dizziness, diplopia, nausea, vomiting, dyspepsia, dry mouth, arthromyalgia, incontinence, impotence	Chronic pancreatitis Pancreatic cancer
Local anesthetics	Procaine 2 g/24 h i.v.	Nausea, vomiting, diarrhea, dizziness, headache, tachycardia, hypertension, rash, severe allergic reactions	Acute pancreatitis
Antidepressants	Duloxetin 30 mg (up to 60 mg) qd.	Headache, drowsiness, dry mouth, nausea, erectile dysfunction, ejaculation disorder, weight loss, constipation, insomnia, vomiting, lack of appetite	Chronic pancreatitis Pancreatic cancer
Cannabinoids	Dronabinol 1 mg (up to 2 mg) qd. Nabilone 1 mg (up to 2 mg) b.i.d.	Psychosis, hallucinations, depersonalization, mood alterations and paranoia, vertigo, drowsiness, dry mouth, ataxia, blurred vision, euphoria	Pancreatic cancer
PERT	Pancrelipase 40,000–50,000 (up to 150,000) IU per meal	Nausea, vomiting, constipation, abdominal swelling, diarrhea	Acute necrotizing pancreatitis with PEI Chronic pancreatitis with PEI Pancreatic cancer with PEI

Abbreviations: b.i.d., twice a day; IU, international unit; i.v., intravenous therapy; NSAIDs, non-steroidal anti-inflammatory drugs; PEI, pancreatic exocrine insufficiency; PERT, pancreatic enzyme replacement therapy; q.i.d., four times a day; qd., once a day; t.i.d., three times a day.

## Data Availability

No new data were created or analyzed in this study. Data sharing is not applicable to this article.
